# A nutrigenomics approach to study the effects of ω-3 fatty acids in laying hens under physiological stress

**DOI:** 10.3389/fphys.2023.1198247

**Published:** 2023-07-25

**Authors:** Atefeh Berenjian, Mohammad Reza Bakhtiarizadeh, Abdollah Mohammadi-Sangcheshmeh, Seyed Davood Sharifi

**Affiliations:** Department of Animal and Poultry Science, Faculty of Agricultural Technology, College of Agriculture and Natural Resources, University of Tehran, Tehran, Iran

**Keywords:** RNA-Seq, nutrigenomics, gene expression, lipid metabolism, stress

## Abstract

Supplement of ω-3 fatty acids can decrease the harmful effects of stress. However, the potential molecular mechanisms that are modulated by dietary ω-3 fatty acids in laying hens under stress remain unknown. Hence, RNA-sequencing (RNA-Seq) technology was used to gain new insights into different gene expression profiles and potential pathways involved in response to stress in the liver of 35-week-old Lohmann LSL-Lite laying hens supplemented with ω-3. Three groups including control (non-stress), stress, and stress_ω-3 fatty acids (three layers per each group) were applied. A total of 1,321 genes were detected as differentially expressed genes of which 701, 1,049, and 86 DEGs belonged to stress vs. control, stress_ω-3 vs. control, and stress vs. stress_ω-3 pairwise comparisons, respectively. Gene ontology and KEGG pathway analysis indicated that the DEGs were enriched in particular regulation of steroid and cholesterol biosynthetic process, fatty acid degradation, AMPK signaling pathway, fatty acid biosynthesis, and immune response. Our data represented a promising approach regarding the importance of ω-3 as anxiolytic and anti-stress. In this context, *UNC13B* and *ADRA1B* genes were downregulated in the stress_ω-3 group compared to the stress group, which are associated with decreased activity of glutamatergic stimulatory neurons and probably play important role in facilitating the response to stress. This study extends the current understanding of the liver transcriptome response to physiological stress, and provides new insights into the molecular responses to stress in laying hens fed a diet supplemented with ω-3 fatty acids.

## 1 Introduction

Laying-type birds reared under commercial conditions are introduced to various stressors such as high density, climate changes, nutritional constraints, fear, infection, and metabolic diseases (Thaxton et al., 2006; Delezie et al., 2007). In addition, domestication and genetic selection for higher egg production and better feed conversion ratio have made birds susceptible to a variety of stresses (Surai and Fisinin, 2016). In the face of stressors, glucocorticoid (GCs) hormones are released from the hypothalamic-pituitary-adrenal (HPA) axis. GCs cause metabolic changes such as the stimulation of hepatic gluconeogenesis, inhibition of glucose uptake by peripheral tissues and suppression of insulin (Romero et al., 2009), mobilization of energy stores, increased lipogenesis and lipid storage (Yuan et al., 2008), inhibition of reproductive activity, and increase in anxiety (Love et al., 2004). GCs can regulate lipid metabolism in adipose tissue, liver, and other organs (Roldan and Herzig, 2015). Genome-wide analysis of glucocorticoid-regulated target genes has shown that the glucocorticoid receptor controls some aspects of the hepatic energy metabolism (Le et al., 2005). It has been reported that more than 50 genes are directly affected by the regulatory actions of GCs (Roldan and Herzig, 2015). In birds, the liver is a central metabolic organ that functions to control the metabolism of glucose and lipid. It is well understood that the liver is a place of some metabolic changes such as gluconeogenesis, changes in lipid composition, and hepatic steatosis which all can be stimulated by GCs. GCs have strong effects on the energy balance of the body, and they are counter-regulatory hormones with broad effects on lipid metabolism. It has been reported that chronic stress reduces docosahexaenoic acid (as an ω-3 fatty acid) content in the phospholipid membranes of glutamatergic neurons at the amygdala, while it increases arachidonic acid content, which in turn inhibits the release of gamma-aminobutyric acid (GABA) from presynaptic neurons (Pérez et al., 2013). Given that, GABA is considered an inhibitory neurotransmitter in the brain. Inhibition of GABA release may increase excitatory neuronal activity in the amygdala, and enhance anxiety. Previous studies reported that ω-3 supplementation increases serotonin levels in the brain of stressed rats (Gross et al., 2002). Serotonin has a vital role in the regulation of anxiety-like behaviors. Studies in humans and mice have revealed the modulating effects of ω-3 fatty acids on the HPA axis and the reduction of glucocorticoid concentrations (Ferraz et al., 2011; Barbadoro et al., 2013). In addition to the anti-stress effects of ω-3 fatty acids in the brain, it has been reported that these compounds reduce the accumulation of triglycerides in the liver through increased and decreased lipid oxidation and triglyceride biosynthesis, respectively (Clarke, 2001). Most of the studies concerning stress in laying hens have been mainly focused on animal performance and biometric traits evaluation and the molecular mechanisms underlying these processes are still unclear. On the other hand, to the author’s best knowledge, the use of ω-3 fatty acids supplementation in stressed birds has never been evaluated for modulating stress. Previous studies have employed reverse transcription polymerase chain reaction (RT-PCR) to explore differentially expressed genes related to the mechanism of action of glucocorticoids and the effects of stress in poultry. In this regard, Wang et al. (2013) suggested that corticosterone decreased the availability of circulating yolk precursors by decreased apolipoprotein synthesis in the liver of laying hens. They also reported the upregulated expression of occludin and claudin in granulosa cells, preventing yolk deposition into oocytes. In another study, Duan et al. (2014) showed that chronic corticosterone administration causes cholesterol accumulation in the pectoralis major muscle of broiler chickens by up-regulating the *HMGCR* mRNA expression. Recently, transcriptome analysis of liver tissue among three distinct genetic chicken lines (heat-susceptible broiler line, heat-resistant Fayoumi line, and their advanced intercross line) suggested that angiopoietin-like 4 is a candidate gene for improvement of heat tolerance in chickens (Lan et al., 2016). Previous studies have evaluated the effects of ω-3 fatty acid on the rodent brain and identified genes involved in neuronal function, stress reactivity, and energy metabolism (Le-Niculescu et al., 2011; Begg et al., 2012). Transcriptome analysis is a powerful approach in functional genomics for a deeper understanding of complicated physiological pathways, such as the lipid metabolism (Bakhtiarizadeh and Salami, 2019). Recently, next-generation sequencing (NGS) has been revolutionizing the field of comprehensive gene expression profiling. In this regard, RNA sequencing (RNA-Seq) is currently the method of choice for performing genome-wide transcriptome analysis. This technology can provide new insights to understand the molecular basis of different biological processes (Bakhtiarizadeh et al., 2019).

To date, little is known about the exact molecular regulatory mechanisms of stress in lipid metabolism. The comprehension of the underlying molecular mechanism is potentially critical to understand the effect of ω-3 fatty acid on stress in birds. Toward this goal, laying hens were used as a model for the study of physiological stress. Then, the effects of physiological stress in laying hens fed by a diet supplemented with ω-3 were assessed to identify differentially expressed genes and explore the underlying molecular regulatory mechanisms. This study can be helpful in identifying candidate genes and molecular pathways involved in response to stress in laying hens.

## 2 Materials and methods

### 2.1 Ethics approval

All methods were carried out in accordance with relevant guidelines and regulations. All experimental protocols were approved by a research council of the University of Tehran.

### 2.2 Trial design

A total of forty-eight 35-week-old Lohmann LSL-Lite laying hens were used in this experiment. Layers were randomly assigned to three trial groups: 1) control (non-stress, no ω-3), 2) stress, birds supplemented by dexamethasone (DEX) (1.5 mg/kg diet), 3) stress_ω-3 fatty acids, stressed birds supplemented with ω-3 (0.48% of diet). Four replicates and four birds per each replicate (16 birds) were considered per each group. DEX is a potent synthetic glucocorticoid and has been suggested in the previous study due to its capability to simulate the effects of the glucocorticoid (Foucaud et al., 1998). Therefore, in the present study, DEX in experimental diets was applied to build a stress model. DEX tablets (Iran Hormone Co., Iran) was dissolved in oil and mixed with a mash diet. Salomega (Agritech, Ireland) was used as an ω-3 fatty acids source to supply the fatty acids desired in the experiment. Salomega is prepared using salmon oil, and corn cob is used as a carrier in this product. It contains 52% fat and about 17% total omega 3 fatty acids. From age of 35–41 weeks, birds were fed by a basal diet supplemented with ω-3 fatty acids. At the beginning of the age of 41 weeks, the stressed birds were continuously fed diets containing DEX (1.5 mg/kg diet) for 1 week (end of 41 weeks). The birds were allocated in 12 cages equipped with 1 drinker and 1 feeder. Cages were 40 × 50 × 35 cm and contained four hens. All birds had *ad libitum* access to water and a basal diet that met all nutritional recommendations of laying hens (Table 1, Lohmann laying hen’s manual). The hens were maintained under similar conditions with (a controlled photoperiod regimen 16:8 h light: dark cycle, temperature 22°C–23°C and relative humidity of 50%–60%). At the end of 41 weeks of age, nine birds (*n* = 3 birds/group) were randomly chosen and were weighed and slaughtered by cervical dislocation. Liver tissue samples were immediately collected, snap-frozen in liquid nitrogen, and stored at 80°C for RNA extraction and RNA-sequencing.

**TABLE 1:** Ingredient and nutrient composition of basal diets.

Ingredient (%)	Control	ω-3 fatty acids
Yellow corn	52.2	49.0
Soybean meal	30.8	31.5
Bran wheat	2.5	2.5
Salomega	0	3
Canola oil	1.83	1.37
Dicalcium phosphate	1.52	1.52
Limestone	6.50	6.50
Oyster shell	3.66	3.66
Vitamin-mineral premix	0.5	0.5
DL-Methionine	0.18	0.16
Sodium chloride	0.15	0.15
Sodium bicarbonate	0.15	0.15
Calculated Nutrients		
AMEn (Kcal/kg)	2,620	2,620
Crude Protein (%)	18.5	18.5
Crude fiber (%)	2.64	2.71
Calcium (%)	4.25	4.25
Available phosphorus (%)	0.43	0.43
Total ω-3 fatty acid (%)	0.031	0.507
Lysine (%)	1.04	1.022
Methionine (%)	0.48	0.44
Methionine + cysteine (%)	0.78	0.74

^a^ Vitamin and mineral Premix supplied per kilogram of diet: Vitamin A, 9000 IU., Vitamin D3, 2000 IU., Vitamin E, 18 IU., Vitamin K3, 2 mg. thiamin, 1.8 mg riboflavin, 6.6 mg. Niacin, 30 mg. Calcium pantothenate, 10 mg. Vitamin B6, 3 mg. Folic acid 1 mg. Vitamin B12, 0.015 mg, Biotin 0.1 mg, Choline 500 mg, manganese oxide 100 mg, ferrous sulfate 50 mg, zinc oxide 100 mg, copper sulphate 10 mg, calcium iodate 1 mg, sodium selenite, 0.2 mg.

### 2.3 Performance

The feed intake, egg number and egg weight were recorded during the stress period, and then the egg production, egg mass, daily feed intake, and feed conversion ratio (FCR) (feed/egg mass) were calculated. All the means of experimental treatments were analyzed by analysis of variance using General Linear Models procedure of SAS (version 9.4). Differences among treatment means were tested using the Tukey test and statistical differences declared at *p* < 0.05.

### 2.4 RNA extraction and transcriptome sequencing

Total RNA was isolated from nine liver samples using TRIzol reagent (Invitrogen, Carlsbad, CA) according to the manufacturer’s protocol. The RNA quality was determined using the Agilent Bioanalyzer 2100 system. The samples were sent to BGI company (Shenzhen, China) to construct a cDNA library and RNA sequencing. Only samples with an RNA integrity number (RIN) greater than 8.5 were used for cDNA library construction. The sequencing was performed on an Illumina HiSeq 2500 platform by paired-end strategy (read length 150 bp).

### 2.5 Quality control and read trimming

The quality of the raw reads was assessed using FastQC (v0.11.5) program to detect common issues in RNA-Seq data. Adapters and low-quality bases were trimmed with Trimmomatic using the options: TRAILING:20, MAXINFO:120:0.9, MINLEN:120. The quality of the reads was re-assessed with FastQC after trimming to confirm quality improvements.

### 2.6 Mapping to genome and identification of differentially expressed genes (DEGs)

Two important steps in RNA-Seq studies are mapping of cleaned reads to the reference genome and normalization and statistical analysis to identify DEGs. In the present study, to increase the accuracy of the identified DEGs our previous pipeline was applied (Bakhtiarizadeh et al., 2019), as the results of two alignment tools (Hisat2 and STAR) and two statistical methods (edgeR and DESeq2) were combined. In this regard, four different combinations including 1) Hisat2 + edgeR, 2) Hisat2 + DESeq2, 3) STAR + edgeR and 4) STAR + DESeq2 were assessed. These tools were selected since they are reliable and robust and also widely used in transcriptome analysis-related studies (Baruzzo et al., 2017; Everaert et al., 2017). Only those genes were considered for further analysis that was identified as DEGs by the four approaches. To do this end, the clean reads were mapped to the reference chicken genome (GRCg6a) using STAR (version 2.5.3a) (Dobin et al., 2013) and Hisat2 software (version 2.1.0) based on the default parameters (Kim et al., 2015). Additionally, HTSeq-count (Python package HTSeq, python v 2.7.3) software was used to count the reads numbers mapped to each gene using the reference annotation file (version 96). Gene expression was quantified as the total number of reads for each sample that uniquely aligned to the reference genome. Differential expression analyses between stress birds *versus* control (S/C), stress_ω-3 *versus* control (S3/C), and stress_ω-3 *versus* stress (S3/S) were performed using two count-based methods in R packages including edgeR (version 3.18.1) (Robinson et al., 2010) and DESeq2 (version 1.16.1). Using a generalized linear model based on the negative binomial distribution. A principal component analysis (PCA) was also employed to assess sample relationships based on voom-transformed values of raw read counts (Law et al., 2014), calculated by the Hisat2+DESEq2 approach.

### 2.7 Gene ontology (GO) and pathway analysis

The DEG lists were submitted to Enrichr web-based tool (Kuleshov et al., 2016) for functional enrichment analysis. This analysis was performed to identify the biological processes and Kyoto Encyclopedia of Genes and Genomes (KEGG) pathways significantly enriched in DEGs. Adjusted *p*-values less than 0.05 were considered as significantly enriched terms.

### 2.8 Real-time PCR analysis

The expression levels of eight DEGs were evaluated by RT-PCR to validate the accuracy of RNA-Seq results, with the RNA samples used for RNA-Seq. Three biological replicates along with two technical replicates were used for each gene. First-strand cDNA synthesis was performed by first-strand cDNA synthesis kit (Thermo Fisher, Co., United States) according to the manufacturer’s guideline. RT-PCR was carried out by Rotor-Gene 6,000 instrument (Corbett Research, Australia) with the Ampliqon Kit (Denmark), according to the manufacturer’s protocol. RT-PCR thermocycling conditions were as follows: an initial 1 cycle at 95°C for 900 s, 40 cycles at 95°C for 15 s, 60°C for 20 s, 72 °C for 20, and a final elongation at 72°C for 1,450 s. All the primer sequences were designed using Primer3Plus software and were blasted against the chicken genome using the national center for biotechnology information (NCBI) Primer-BLAST software to guarantee unique production. The genes and related primer sequences are listed in Table 2. CT-method was applied to quantify changes in gene expression and *GAPDH* and *18s rRNA* genes were used as reference genes for the normalization of gene expression levels. The relative gene expression values were calculated using the Delta-Delta Ct method and calculated fold changes were compared with RNA-Seq results.

**TABLE 2:** Genes and related primers for RT-PCR analysis.

Genes	Primer sequence	Product size (bp)
*SQLE*	F: TGT​GTC​TCA​GGT​CCT​GTT​GG	120
	R: CCA​CGA​CTC​CGA​CTT​AAA​GC	
*CYP51A1*	F: TCA​CTA​TGG​TTG​GCA​AGA​CG	126
	R: CAA​AGA​CTG​GTG​TGG​TCA​GC	
*SCD*	F: ACC​ACA​AGT​TCT​CCG​AGA​CG	99
	R: CAT​CTG​GGT​GTT​TGC​GTA​CC	
*APOC3*	F: CTC​CCG​ATA​AGA​CAG​AAG​TGG	95
	R: CTC​ATG​CAC​TGT​GGT​GAA​GG	
*ACOX1*	F: ATT​CGT​CCC​AAT​GCA​GTA​GC	92
	R: TAC​ACG​TTG​CCA​TCA​TAC​CG	
*PPARGC1A*	F: GTA​AAT​TTG​CGG​GAT​GAT​GG	139
	R: TGC​GTC​CAC​AAA​AGT​ACA​GC	
*CPT1A*	F: TGT​GGC​TGA​TGA​TGG​TTA​CG	113
	R: TTC​CAA​AGC​GAT​GAG​AAT​CC	
*MSMO1*	F: TCT​TTC​CTG​CCT​GAC​AAT​CC	90
	R: CGT​CGC​TAT​CTG​GAA​CTT​GG	
*GAPDH*	F: ACA​TGG​CAT​CCA​AGG​AGT​GAG	144
	R: GGG​GAG​ACA​GAA​GGG​AAC​AGA	
*18SrRNA*	F: ATA​ACG​AAC​GAG​ACT​CTG​GCA	136
	R: CGG​ACA​TCT​AAG​GGC​ATC​ACA	

## 3 Results

### 3.1 Laying performance

The effects of stress and dietary ω-3 fatty acids supplementation on the performance of laying hens are shown in Table 3. Body weight were not affected (*p* > 0.05) by the trial treatments. However, it tended to be decreased in response to stress.

**TABLE 3:** Effects of Trial treatments on laying performance in laying hens.

Item	Live body weight (g)	Egg production (%)	Egg mass (g/hen/day)	Feed intake (g/hen/day)	FCR
Control	1439.33	97.91^a^	61.14^a^	114.04^a^	1.86
Stress	1254.66	91.96^a^	56.24^b^	101.46^b^	1.80
Stress_ω-3 fatty acids	1229.33	85.71^b^	53.08^b^	96.11^c^	1.81
SEM^d^	56.26	1.89	1.15	1.56	0.03
*p*-value	0.07	0.004	0.002	<0001	0.41

^a–c^ Means with different superscripts within a column are significantly different at *p* < 0.05.

^d^ SEM: standard error of means.

Induction physiological stress reduced feed intake, egg production and egg mass (*p* < 0.05). Stressed birds that fed on diet containing ω-3 fatty acids had lower feed intake, egg production and egg mass compared to the other groups. Trial tretments had no significant effect on FCR (*p* > 0.05).

### 3.2 Alignment and mapping of the chicken genome

To investigate the molecular mechanisms involved in response to the physiological stress of laying hen fed by ω-3 fatty acids, three treatment groups including control, stress, and stress_ω-3 were considered and cDNA libraries from nine chicken liver tissues (three per treatment group) were constructed for high throughput RNA sequencing. RNA-Seq generated around 219,189,680 paired-end reads ranging from 24,023,561 to 24,614,348, with an average of 24,273,029 in control, 24,411,997 in stress and 24,378,201 in stress_ω-3 treatment group. For control, stress, and stress_ω-3 groups, an average of 24,272,851, 24,411,859, and 24,378,088 reads per sample passed the quality control. On average, 85% and 79% of all the clean reads were aligned to the chicken genome by Hisat2 and STAR tools, respectively. Detailed information on raw and clean reads along with mapping results are presented in Table 4.

**TABLE 4:** Summary of the generated reads and their aligning to the reference genome.

Treatment group	Raw reads	Trimmed reads	Hisat2 total mapping (%)	STAR total mapping (%)	Hisat2 uniquely mapped reads (%)	STAR uniquely mapped reads (%)
Control _1	24,332,749	24,332,563	20,966,772 (86)	19,218,145 (79)	20,204,469 (83)	18,573,551 (76)
Control _2	24,260,559	24,260,353	20,658,892 (85)	19,061,873 (79)	19,902,808 (82)	18,421,764 (76)
Control _3	24,225,780	24,225,637	21,076,475 (87)	19,435,695 (80)	20,285,212 (84)	18,778,552 (78)
Stress_1	24,598,081	24,597,837	21,125,849 (86)	19,333,339 (79)	20,337,395 (83)	18,688,218 (76)
Stress_2	24,023,561	24,023,434	20,816,290 (87)	19,121,020 (80)	20,030,311 (83)	18,474,302 (77)
Stress_3	24,614,348	24,614,307	21,490,698 (87)	20,130,300 (82)	20,742,986 (84)	19,529,573 (79)
Stress_ω-3_1	24,404,896	24,404,664	20,013,528 (82)	18,216,548 (75)	19,362,709 (79)	17,648,474 (72)
Stress_ω-3_2	24,612,476	24,612,423	20,267,539 (82)	18,681,744 (76)	19,626,912 (80)	18,064,359 (73)
Stress_ω-3_3	24,117,230	24,117,177	20,311,874 (84)	18,602,650 (77)	19,620,770 (81)	17,988,398 (75)

### 3.3 Differentially expressed genes

**FIGURE 1:**
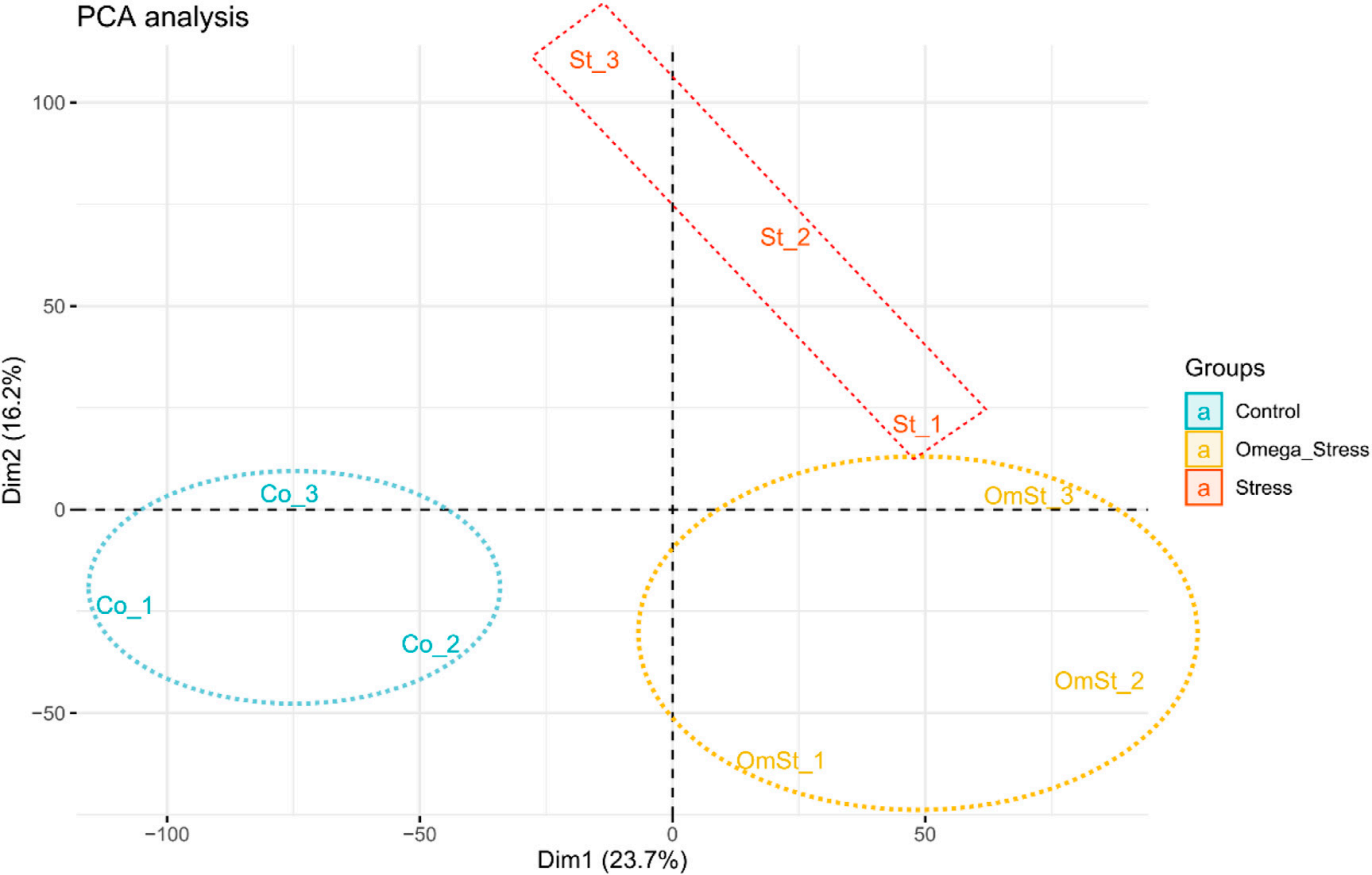
PCA plot of gene expression profiles of the treatments. This plot shows the variance of the three biological replicates of each of the three treatments. The percentages on each axis represent the percentages of variation explained by the principal components.

Across all samples, a total of 16,595 genes were identified to be expressed (read counts >0), accounting for 68% of the total annotated genes. PCA analysis was performed to check the level of similarity/dissimilarity in the gene expression profiles of the three treatments. This analysis shows if samples from the same treatment cluster together or not. Results of this analysis based on the first two principal components showed that all the samples can be divided into three clusters according to their treatments (Figure 1). Hence, the differences in the transcriptome profiles can be appropriate for detecting putative candidate genes explaining the known differences that exist among the treatments.

**FIGURE 2:**
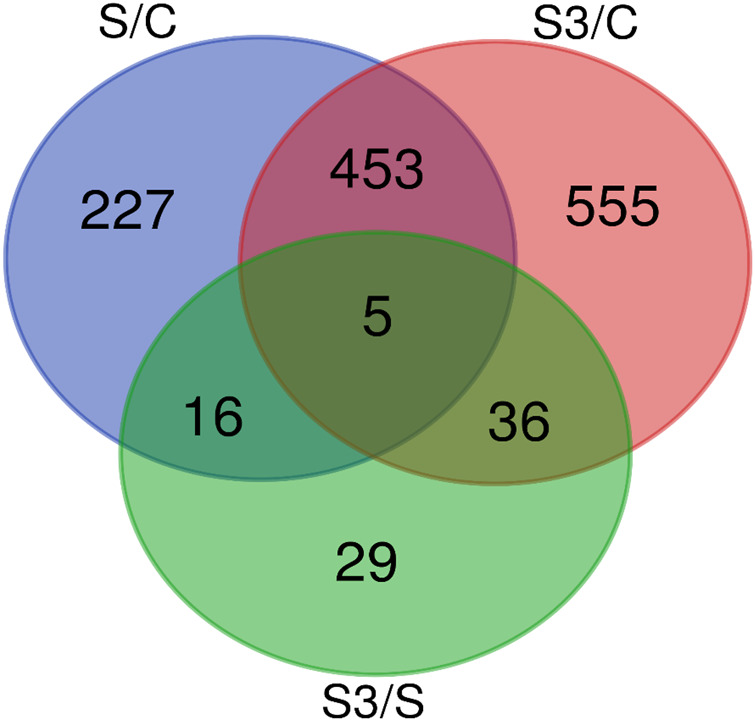
Venn diagram depicting shared DEGs among pairwise comparisons.

To explore the transcriptional changes of the genes affected by the applied treatments, DEGs were identified by three pairwise comparisons using two well-established statistical analysis methods including DESeq2 and edgeR. Therefore, four approaches were used to identify DEGs: Hisat2 + edgeR, Hisat2 + DESeq2, STAR + edgeR and STAR + DESeq2. A total of 1,321 DEGs were identified among the three comparisons; the majority of DEGs were generated in S/C and S3/C comparisons. The comparative profile of the DEGs in all the comparisons revealed that more numbers of DEGs were downregulated. A total of 701 DEGs were detected in S/C, with 302 upregulated and 399 downregulated genes in the stress group. A total of 1,049 DEGs were identified in S3/C, with 428 upregulated and 621 downregulated genes in the stress ω-3 group. Only 86 DEGs were observed in S3/S, with 31 upregulated and 55 downregulated genes in the stressed ω-3 group. The shared DEGs among pairwise comparisons are depicted as Venn Diagrams in Figure 2. There was a significant increase in DEGs detected in both S/C and S3/C comparisons than S/S3. Furthermore, the highest number of common DEGs were detected in the comparison of the control group against under-stress treatments (453 genes). Only five genes were commonly identified as DEGs in all comparisons including calcium-regulated heat stable protein 1 (*CARHSP1*), discoidin CUB and LCCL domain containing 1 (*DCBLD1*), acetyl-CoA carboxylase alpha (*ACACA*), macrophage stimulating 1 receptor (*MST1R*) and cytochrome P450, family 1, subfamily A, polypeptide 2 (*CYP1A2*).

### 3.4 Functional enrichment analysis

**FIGURE 3:**
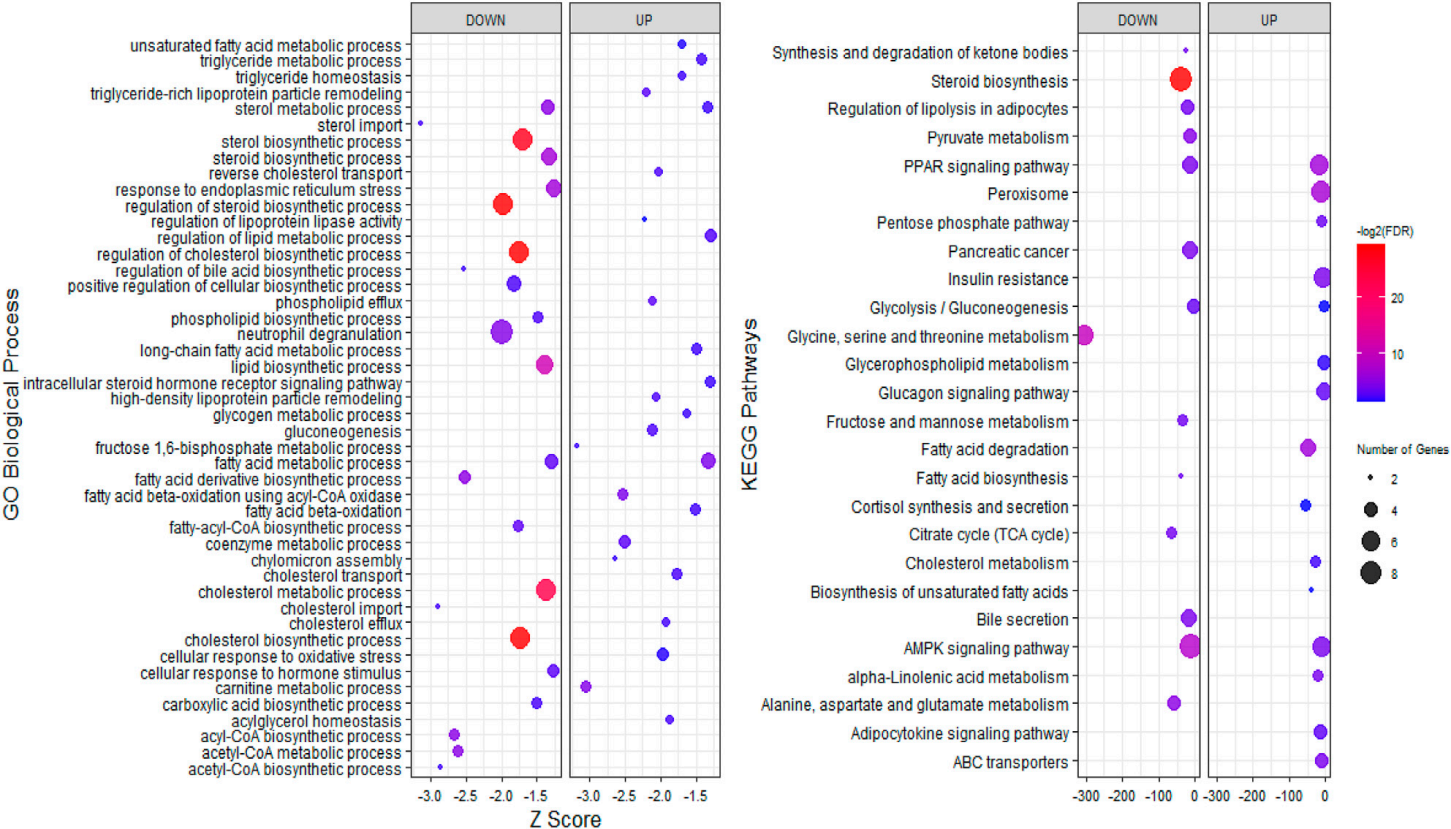
GO and KEGG pathway analysis of upregulated and downregulated DEGs S/C comparison.

**FIGURE 4:**
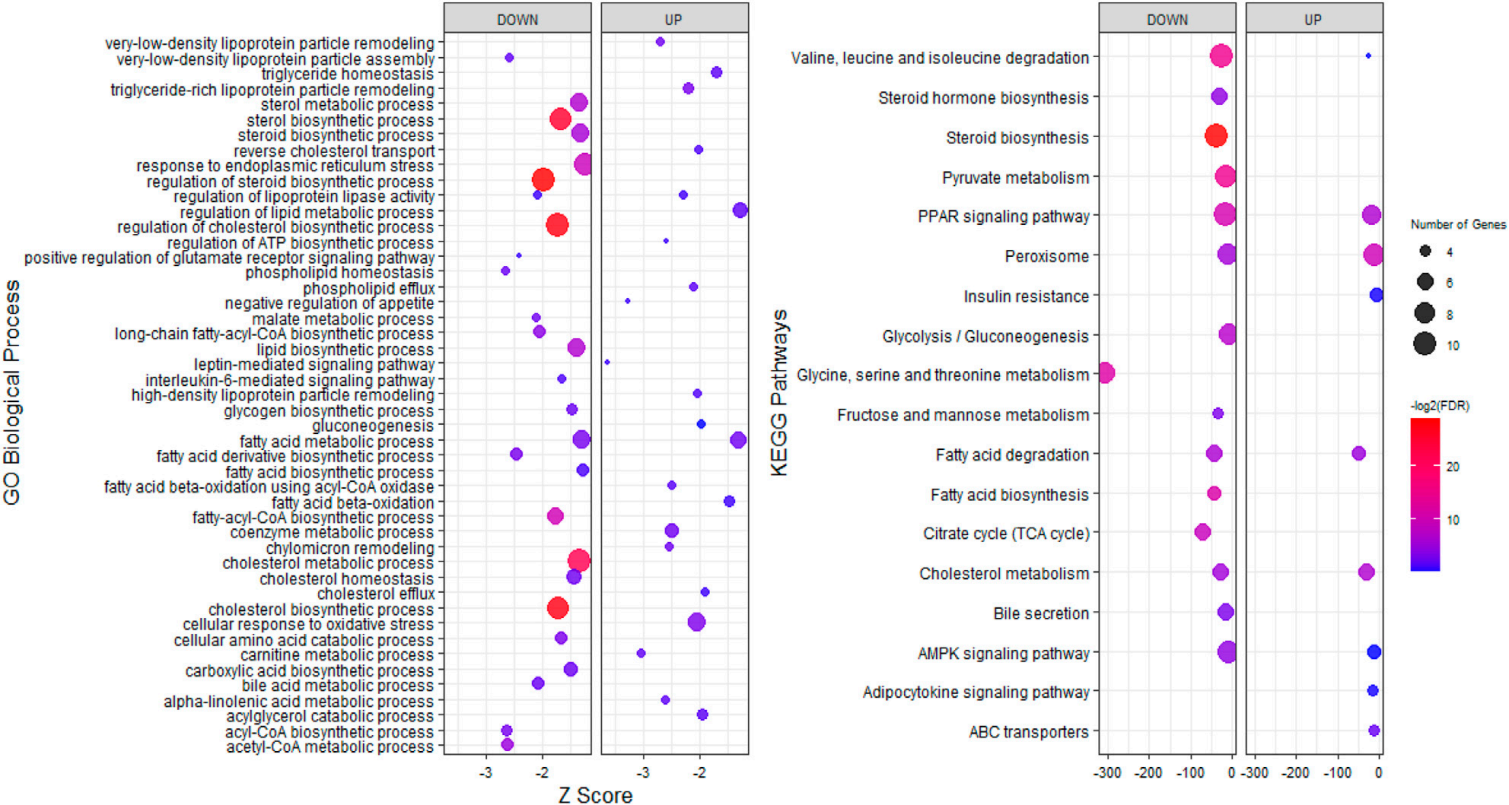
GO and KEGG pathway analysis of upregulated and downregulated DEGs S3/C comparison.

**FIGURE 5:**
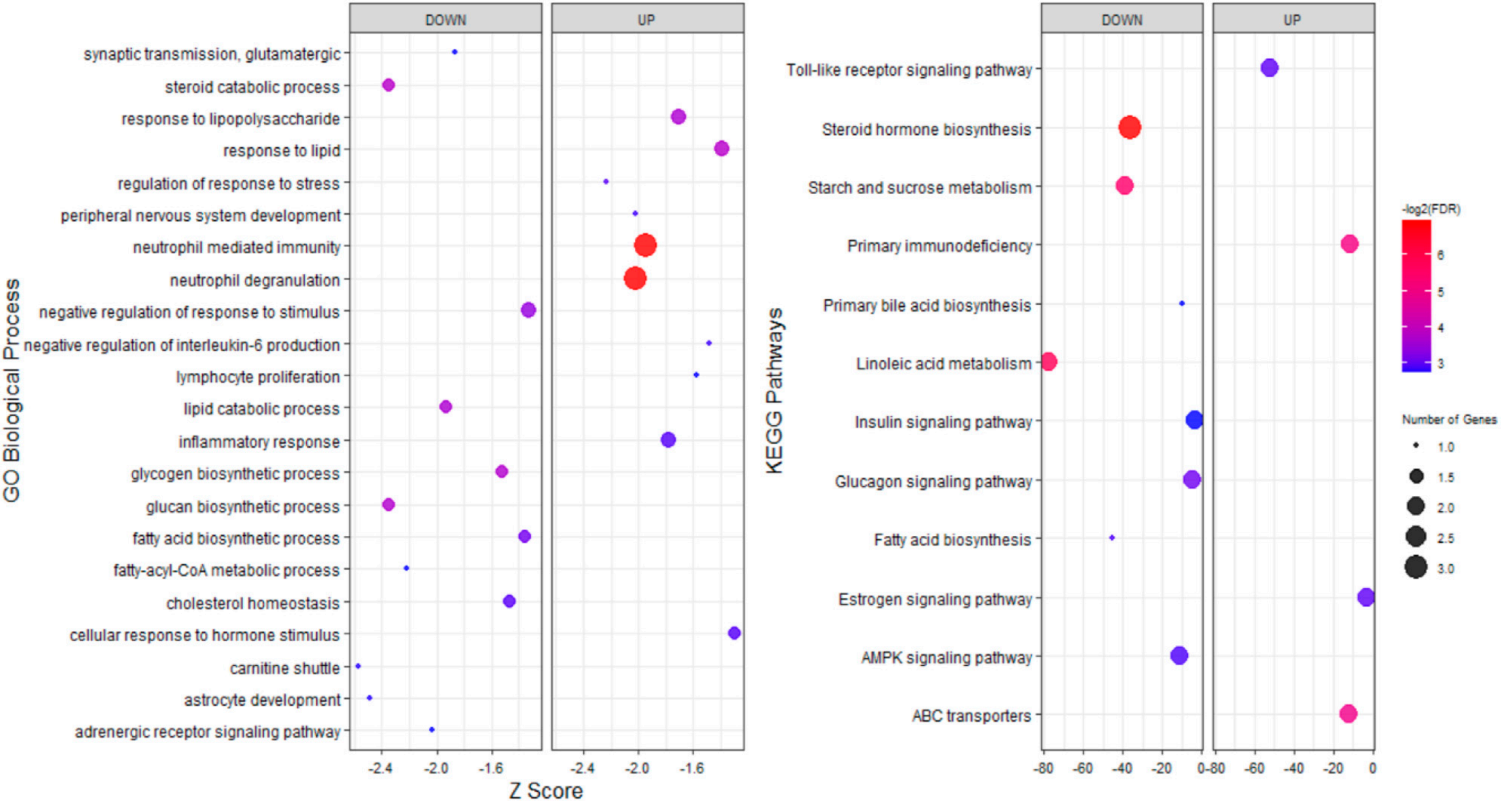
GO and KEGG pathway analysis of upregulated and downregulated DEGs S/S3 comparison.

To explore the potential functions of stress-responsive genes, DEGs from each of the pairwise comparisons were subjected to functional enrichment analysis, as upregulated and downregulated DEGs were treated separately. Comparing the stress and control groups of genes with different expressions, it was observed that 302 upregulated DEGs were significantly enriched in 117 biological processes (BP) terms and 22 KEGG pathways, while 399 downregulated DEGs were significantly enriched in 212 BP terms and 38 KEGG pathways (Supplementary File S1). The most important enriched BP terms and KEGG pathways are shown in Figure 3. When comparing the stress_ω-3 and control groups, 428 upregulated and 621 downregulated DEGs were enriched significantly in 149 and 223 BP terms and 11 and 30 KEGG pathways, respectively (Supplementary File S2). Figure 4 indicates the most notably enriched BP terms and KEGG pathways. Finally, 31 upregulated and 55 downregulated DEGs were enriched in102 and 136 BP terms as well as 13 and 12 KEGG pathways, respectively (*p* < 0.5, Supplementary File S3), according to the comparison between stress_ω-3 and stress groups. The considerably enriched BP terms and KEGG pathways are shown in Figure 5. Most of the significant terms or pathways in control against under stress groups comparisons were related to lipid metabolism such as fatty acid oxidation, cholesterol biosynthetic process, biosynthesis of unsaturated fatty acids, steroid biosynthesis, fatty acid biosynthesis, reverse cholesterol transport, acyl-CoA biosynthetic process, and cholesterol efflux. Other important GO terms and KEGG pathways enriched in insulin resistance, AMPK signaling pathway, cortisol synthesis and secretion, steroid hormone biosynthesis, cellular response to oxidative stress, bile secretion, and peroxisome included. The functional analysis of S/S3 indicated that most of the upregulated DEGs were significantly enriched in immune response-related biological processes or pathways, while most downregulated DEGs were enriched in lipid and glucose metabolism as well as neurotransmission biological processes or pathways. Some important enriched terms related to immune response and nervous system were lymphocyte proliferation, negative regulation of interleukin-6 production, negative regulation of interleukin-6 production, peripheral nervous system development and synaptic transmission, and glutamatergic. Taken together of all functional enrichment analysis results, the following biological processes and KEGG pathways were the most affected: cholesterol metabolism, ATP-binding cassette (ABC) transporters, insulin resistance, AMPK signaling pathway, fatty acid degradation, and sterol metabolic process.

### 3.5 Validation of differentially expressed genes by RT-PCR

**FIGURE 6:**
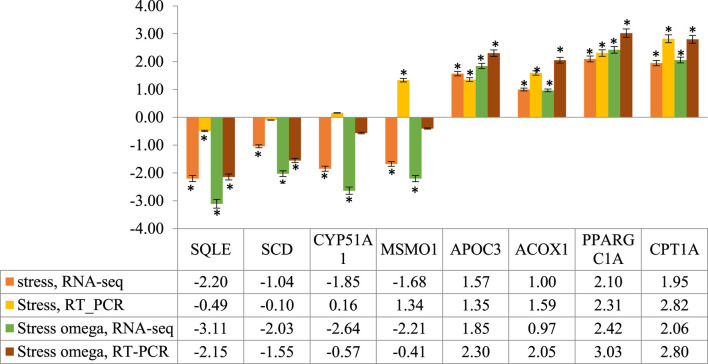
RT-PCR validation of the eight randomly selected genes identified by RNA-Seq analysis in stress and stress-ω-3 groups. *The differences between experimental groups are significant at *p* < 0.05.

To confirm the results obtained from RNA-Seq, eight DEGs were randomly selected from both upregulated (*SQLE*, *SCD*, *CYP51A1*, and *MSMO1*) and downregulated (*APOC3*, *ACOX1*, *PPARGC1A*, and *CPT1A*) genes. Although fold changes varied between the two methods, however, all the genes showed a similar expression pattern between RNA-Seq and RT-PCR results, except *MSMO1* and *CYP51A1* in the S/C comparison (Figure 6). Therefore, RT-PCR and RNA-Seq results were in good agreement, indicating the reliability of the RNA-Seq data.

## 4 Discussion

The combination of ω-3 fatty acids and stress reduced feed intake in stressed birds. Decreased feed intake and performance in laying hens and quails has been reported previously following the treatment with corticosterone and dexamethasone (Liu et al., 2012; Berenjian et al., 2018). Activation of the HPA axis as a result of stressors can causes the secretion of the corticotropin-releasing factor (CRF), which is a powerful anorexigenic peptide in birds (Richardson et al., 2000). Gray et al. (2013) reported that ω-3 fatty acids increase leptin levels and reduce appetite in obese patients. On the other hand, Houseknecht et al. (1998) stated that cortisol hormone stimulates the levels of leptin expression.

The feed intake in birds under stress can be diminished by the increased level of leptin as a satiety hormone that is created by the use of ω-3 fatty acids. In general, the synergistic effects of stress and ω-3 fatty acids reduce feed intake of birds. The adverse effects of stress on laying performance investigating by the present paper, are in agreement with other researches (Wang et al., 2013). The reduced productive performance in this study can primarily be due to the reduced feed intake. While reducing feed intake, the energy required to produce yolk precursors in the liver is not provided; resulting in the suppressed development of follicles and ovulation by reducing the availability of yolk precursors. The cumulative effect of stress and omega-3 fatty acids could reduce the feed intake, and the use of omega-3 fatty acids could not improve the negative effects of stress on laying performance as tested at dosage in this research.

The action of GCs involves a high diversity of metabolic effects, such as the stimulation of hepatic gluconeogenesis, proteolysis, and changes in lipid metabolism. High metabolic rate due to GCs has been hypothesized to result in elevated oxidative stress (Lin et al., 2004; Caro et al., 2007). In this study, genes related to lipid and glucose metabolism in the liver were significantly activated by stress induced by DEX. It shows that GCs play an important role in energy metabolism by regulating the genes involved in the metabolic pathways, including the lipid metabolism (Roldan and Herzig, 2015; Hu et al., 2019).

Here, several pathways and biological processes involved in lipid metabolism were enriched in the liver tissue of laying hens under stress. These pathways included cholesterol and steroid biosynthesis, cholesterol metabolism, peroxisome pathway, fatty acid degradation, AMPK signalling pathway, insulin resistance, ABC transporters, and glucagon signalling pathway. Most of the significantly enriched terms were observed in S/C and S3/C comparisons than in S3/S, indicating larger differences in the stress-related treatments transcriptome profiles compared with control conditions, which was supported by the more DEGs in control against the two other treatments.

Cholesterol is an important factor in the biosynthesis of steroids and steroid hormones and is closely related to lipid metabolism. Seven genes were associated with the regulation of cholesterol and steroid biosynthetic process including sterol regulatory element-binding proteins 1/2 (*SREBP1/2*), 3-hydroxy 3-methyl glutaryl-CoA reductase (*HMGCR*), fatty acid synthase (*FASN*), squalene epoxidase (*SQLE*), stearoyl-CoA desaturase (*SCD*) and cytochrome P450 family 51subfamily A member 1 (*CYP51A1*), which were downregulated in stressed birds (stress and stress_ω-3 groups) compared to control birds. SREBPs are located in the center of cholesterol homeostasis. They are a family of transcription factors anchored in the endoplasmic reticulum membrane. SREBPs are responsible for maintaining cholesterol homeostasis, acting on synthesis and uptake in response to the cholesterol depletion (Shimano et al., 1996). *SREBP1* plays a key role in the regulation of lipogenesis in birds (Assaf et al., 2003). *SREBP2* increases the expression of the *HMGCR* gene, which is a rate-limiting enzyme in the cholesterol synthesis (Maxfield and Tabas, 2005). *SCD* is a microsomal enzyme that catalyzes the synthesis of monounsaturated fatty acids (MUFAs) from saturated fatty Acyl-CoAs in the liver tissue (Lefevre et al., 1999). *SQLE* is an enzyme located in the endoplasmatic reticulum catalyzing the epoxidation of squalene and producing 2,3-oxidosqualene. *SQLE* is one of the rate-limiting enzymes in the cholesterol biosynthesis (Ono and Imai, 1985). According to the results of this study, decreased expression of SREBPs as transcription factors could reduce the expression of genes involved in lipid and cholesterol synthesis.

*CYP51A1* catalyzes the methylation of lanosterol to dimethyl colostrum, the main pathway in the biosynthesis of sterols (Lewińska et al., 2013). In the present study, physiological stress and ω-3 fatty acids reduced the synthesis of cholesterol, steroids, and fatty acids by reducing the expression of the above genes. It was shown that the expression of *FASN* is regulated by the glucocorticoids (Xu et al., 2009). As shown above, feed intake was reduced in stressed hens and they were exposed to decrease accesses of energy. Reduce energy intake leads to the breakdown of body reserves such as lipids and proteins to produce ATP, while processes like lipid synthesis are limited. However, contrary to this result, the expression of genes involved in cholesterol biosynthesis and fatty acids such as *SREBP*s, *SCD*, *FASN*, *HMGCR*, and *CYP51A1* in laying hens and broilers increased by GCs (Cai et al., 2011; Roldan and Herzig, 2015; Hu et al., 2019). Similar to the results of this study, a reduction in lipid synthesis and accumulation in mice fed with a diet supplemented with ω-3 fatty acids has been previously reported (Valenzuela et al., 2012). ω-3 fatty acids downregulate the expression of transcription factor *SREBP-1* with inhibition of the transcription of lipogenic genes such as *FASN*, acetyl-CoA carboxylase, stearoyl-CoA desaturase-1, which can be led to a decrease in *de novo* lipogenesis (Valenzuela et al., 2012). Based on the findings of this study, both of stress and ω-3 fatty acids factors reduced lipid synthesis and accumulation in the liver by decreasing the expression of *SREBP* as the main regulator of lipogenesis in birds.

The next important enriched pathway was cholesterol metabolism. Some important genes in this pathway were cholesteryl ester transfer protein (*CETP*), apolipoprotein A1 (*APOA1*), and apolipoprotein C3 (*APOC3*), which were all upregulated in under stress treatments (S and S3 groups) compared with the control group. These genes play a role in the reverse transport and removal of cholesterol from tissues to the liver (Bolanos-Garcia and Miguel, 2003). In line with these results, the expression of the *APOA1* gene was increased in glucocorticoid treatment, which is the main protein involved in HDL particles (Taylor et al., 1996). In fact, increasing the reverse transport of cholesterol to the liver is a response to the decrease in cholesterol synthesis in stressed birds.

The next pathway was fatty acid degradation-related genes such as carnitine palmitoyl transferase 1A (*CPT1A*), acyl CoA oxidase 1 (*ACOX1*), and acetyl CoA acyltransferase 1 (*ACAA1*), which were all upregulated in the stressed groups compared to control group. *CPT1A* is a rate-limiting enzyme associated with β-oxidation of fatty acids in mitochondria and has a dominant role in the triglyceride metabolism (Zammit, 2008; Akkaoui et al., 2009). *CPT1A* upregulation increases the oxidation of fatty acids in hepatocytes and reduces the lipid accumulation (Akkaoui et al., 2009). *ACOX1* is a flavoenzyme that catalyzes the initial and rate-determining reaction of β-oxidation of fatty acids in the peroxisome, *ACOX1* catalyzes the desaturation of acyl-CoAs to 2-trans-enoyl-CoAs (Zeng et al., 2017). *ACAA1* encodes an enzyme that cleaves 3-ketoacyl CoA to give acetyl-CoA and acyl-CoA during the fatty acid β-oxidation in the peroxisome (Li et al., 2019). Activation of fatty acid oxidation in stressed birds, shift the function of the liver toward increasing energy supply and reducing anabolism. Dietary supplementation of ω-3 fatty acids in stressed birds also increased oxidation of fatty acids and decreased lipid accumulation in tissue. In line with these views, enhanced fatty acid oxidation was observed in the liver of mice supplemented with ω-3 fatty acids (Breuner and Hahn, 2003).

ABC transporters such as ATP binding cassette subfamily B member 1 (*ABCB1*), ATP binding cassette subfamily A member 12 (*ABCA12*), and ATP binding cassette subfamily G member 2 (*ABCG2*) were upregulated in under stress groups than the control (*p* < 0.05). The ABC transporters are efflux transporters and play an important role in the absorption, distribution, excretion, and toxicity of the xenobiotics (Tlemsani et al., 2015). Moreover, several genes involved in the AMPK signaling pathway and insulin resistance such as *CPT1A*, PPARG coactivator 1 alpha (*PPARGC1A*), glucose-6-phosphatase catalytic subunit (*G6PC*), and glycogen synthase 2 (*GYS2*) were upregulated, while *SREBP1*, Acetyl-CoA carboxylase alpha (*ACACA*), insulin receptor substrate 1 (*IRS1*), *SCD*, *HMGCR* and *FASN* in AMPK signaling pathway were downregulated in the under-stress birds compared with the control group. *G6PC* is involved in the final stages of hepatic glucose production. *GYS2* is the inactive form of glycogen synthase, which reduces glycogen synthesis. AMPK is the main sensor of cellular energy status that is activated in response to stress to restore energy balance by inhibiting ATP-consuming pathways and expanding ATP-producing processes. In this regard, *SREBP1* a transcriptional regulator of the genes involved in lipid synthesis, biosynthesis, and uptake of cholesterol, is inhibited by AMPK (Li et al., 2011). Similar to our results, it was observed that activation of the AMPK pathway in mammals stimulated hepatic fatty acid oxidation and decreased cholesterol synthesis and *De novo* lipogenesis (Winder and Hardie, 1999). Also, in rats, the administration of dexamethasone promoted acetyl-CoA carboxylase phosphorylation and increased oxidation of fatty acids, likely by activating the AMPK (Qi et al., 2006).

Insulin resistance was the other activated pathway in birds under stress. Rare mutations of the insulin receptor gene (Baynes et al., 1997) and IRS1 protein (Yoshimura et al., 1997) lead to insulin resistance. It has been shown that high concentrations of hormones such as cortisol, glucagon, and catecholamines are associated with impaired insulin action (Reynolds and Walker, 2003). Insulin resistance induced by GCs can result from an alteration in the molecules involved in the insulin signaling cascade. Glucose intolerance, high blood pressure, and insulin resistance are common in people with high blood cortisol levels (Reynolds and Walker, 2003). Decreased expression of the *IRS1* gene in this study, could indicate insulin resistance in laying hens under physiological stress. Consistent with these results, chronic treatment with corticosterone decrease IRS-1 and insulin receptor and evokes insulin resistance in chickens (Dupont et al., 1999). Based on the results of this study, it can be stated that activation of AMPK and insulin resistance pathways, as a result of stress, as well as, the act of ω-3 fatty acids as signaling molecules regulating hepatic lipid metabolism, prevented lipogenesis in liver tissue, whereas, increased oxidation of fatty acids.

These results are in contrast to various experiments and increased lipogenesis, cholesterol synthesis, and hepatic steatosis as a result of the use of GCs or a variety of stresses in different birds. The difference in results can be explained by the fact that the AMPK modulates glucocorticoid action in the target tissues. On the other hand, insulin resistance induced by dexamethasone is associated with several changes along the insulin signaling cascade. Because insulin signaling is required for lipogenic activity of the glucocorticoid in the liver, activation of AMPK and insulin resistance pathways prevented lipogenesis and hepatic steatosis in laying hens under stress.

Cytochrome P450 family 17 subfamily A member 1 (*CYP17A1*) and phosphodiesterase 8A (*PDE8A*) are involved in cortisol synthesis and secretion. These genes were upregulated in the liver of stressed birds compared to that in the liver of control birds (S/C comparison). Stress leads to the secretion of stress hormone and subsequently challenge the patterns of physiological response. The *CYP17A1* is an important enzyme in the pathway of steroid biosynthesis and converts pregnenolone to 17-hydroxy pregnenolone (Zhang et al., 2019). *PDE8A* modulates corticosterone secretion in the adrenal gland (Tsai and Beavo, 2011). These findings are in good agreement with previous works as increased synthesis of corticosterone in the birds under stress is reported (Post et al., 2003; Xie et al., 2015).

Genes of acetyl-CoA carboxylase alpha (*ACACA*) and GYS2 were enriched in the KEGG pathways of AMPK, insulin, and glucagon signaling. These genes were downregulated in a stress ω-3 group compared to the stress group. The fatty acid biosynthesis pathway with one gene (*ACACA*) was also downregulated in this comparison. *ACACA* converts acetyl-CoA to malonyl-CoA in fatty acid biosynthesis. These results are similar to the results of comparisons of stressed birds *versus* control that were previously stated. These findings confirm the role of ω-3 fatty acids in reducing lipid biosynthesis.

Furthermore, we observed increased gene expression of neurofascin (*NFASC*) and decreased genes expression of unc-13 homolog B (*UNC13B*) and adrenoceptor alpha 1B (*ADRA1B*) in the stress_ω-3 group compared to the stress group. Neurofascin is a transmembrane protein that plays an essential role in nervous system development and function of the axon initial, encoded by *NFASC* gene (Monfrini et al., 2019). *ADRA1B* is involved in the adrenergic receptor signaling pathway and plays an important role in facilitating the response to the HPA axis during stress. Stimulation of *ADRA1B* is mainly associated with decreased sodium and potassium conduction and slow depolarization (Bergles et al., 1996). In agreement with these results, the use of dexamethasone in cultured smooth muscle cells increased the expression of the *ADRA1B* gene (Sakaue and Hoffman, 1991). *UNC13B* is a presynaptic protein that promotes the priming of synaptic vesicles by acting through syntaxin (Söllner, 2003). *UNC13B* is involved in the biological term of synaptic transmission, glutamatergic. *UNC13B* downregulation is associated with decreased activity of glutamatergic stimulatory neurons. Glutamate is a stimulant neurotransmitter in the hippocampus. Increased release of glutamate from pre-synapse neurons has been reported in rodents under chronic stress (Fontella et al., 2004). Chronic corticosterone administration in rats also increased the release of glutamate in the synaptic space (Wang and Wang, 2009). The use of ω-3 fatty acids in this experiment reduced the expression of the genes beta-adrenergic receptors and consequence the glutamatergic signaling pathway decreased. In this regard, it was reported that supplementation of diet with ω-3 fatty acids decreases neurotransmitter release by presynaptic neurons and glutamate receptors in both rodents and humans subjected to stressful situations (Hennebelle et al., 2014). Latour et al. (2013) showed that the supplementation of ω-3 fatty acids improves the uptake of glutamate by astroglia, and in this way, prevents damage and death of neurons due to the accumulation of glutamate in synapses. The present findings suggest that ω-3 fatty acid supplementation decreases glutamatergic activity by reduction of glutamate receptor genes expression. Previous studies have reported that ω-3 fatty acids have anti-stress effects and reduce anxiety and depression in humans and rat (Takeuchi et al., 2003; Pérez et al., 2013). Therefore, according to these results, the use of ω-3 fatty acids in laying hens might have anti-stress effects on this animal.

## 5 Conclusion

RNA-Seq was applied to identify DEGs, significantly enriched biological processes, and KEGG pathways in laying hens under physiological stress, which were fed a diet supplemented with ω-3 fatty acids. Our data demonstrated a marked effect of stress on the regulation of genes and pathways involved in hepatic lipid metabolism. The DEGs between stress and stress_ω-3 groups *versus* control were enriched for genes involved in the peroxisome, fatty acid degradation, insulin resistance, AMPK signaling pathway, cholesterol metabolism, and steroid biosynthesis. Activation of the above routes in particular AMPK, insulin resistance pathways, and oxidation of fatty acids indicate a decrease in lipogenesis and lipid accumulation in liver tissue. In other words, both factors, namely, DEX-induced stress in stressed groups and the use of ω-3 fatty acids in the stress_ω-3 group could reduce the synthesis of lipid and increase fatty acid oxidation. Furthermore, the *NFASC*, *UNC13B*, and *ADRA1B* involved in the adrenergic receptor signaling pathway were differentially expressed in the stress_ω-3 group compared to the stress group. It appears that ω-3 fatty acid supplementation may ameliorate dexamethasone-induced stress by reducing activity in the glutamatergic pathway. Further research needs to be conducted to provide a better understanding of the beneficial effects of omega-3 to alleviate the adverse effects of stress on poultry.

## Data Availability

The datasets presented in this study can be found in online repositories. The names of the repository/repositories and accession number(s) can be found in the article/Supplementary Material.
